# Cardiopulmonary resuscitation of a very preterm infant using high-frequency oscillation ventilation

**DOI:** 10.1016/j.resplu.2022.100265

**Published:** 2022-06-28

**Authors:** Julia Buchmayer, Lukas Wisgrill, Michael Schneider, Tobias Werther, Katharina Goeral, Angelika Berger, Georg M. Schmölzer, Michael Wagner

**Affiliations:** a Comprehensive Center for Pediatrics, Department of Pediatrics and Adolescent Medicine, Division of Neonatology, Intensive Care and Neuropediatrics, Medical University of Vienna, Vienna, Austria; b Centre for the Studies of Asphyxia and Resuscitation, Neonatal Research Unit, Royal Alexandra Hospital, 10240 Kingsway Avenue NW, Edmonton, AB T5H 3V9, Canada; c Department of Pediatrics, University of Alberta, Edmonton, Canada

**Keywords:** Newborn, Preterm, Delivery room, Neonatal resuscitation, High-frequency oscillation ventilation, Cardiopulmonary resuscitation, **CPR**, cardiopulmonary resuscitation, **HFOV**, high-frequency oscillation ventilation, **MAP**, mean airway pressure, **PPV**, positive pressure ventilation, **ROSC**, return of spontaneous circulation

## Abstract

We present a novel approach of ventilation, using high-frequency oscillation ventilation (HFOV), during neonatal cardiopulmonary resuscitation (CPR) of a very preterm neonate. This case report highlights the importance of adequate lung inflation, which is a current topic, with neonatal resuscitation guidelines recommending a coordinated 3:1 compression:ventilation ratio during CPR. Our patient, a female infant born at 30 weeks gestational age, weighing 970 g, appeared floppy and apneic following birth in the amniotic sac. Lungs were unfolded and white-out in an x-ray done during resuscitation. The aim was to open lungs effectively using HFOV, instead of positive pressure ventilation, which was used unsuccessfully until the 7th minute of life. Heart rate continuously dropped below 60/min 15 min after birth and chest compressions with asynchronous HFOV were started, adrenalin was administered three times and surfactant was instilled endotracheally twice. It was possible to stabilize the patient after 15 min of CPR, following return of spontaneous circulation. HFOV may have enabled an alternative and rescue option of ventilation during neonatal CPR in this case.

## Introduction

The case describes the novel use of high-frequency oscillation ventilation (HFOV) during neonatal resuscitation and highlights the controversial topic of the coordination of ventilation and chest compression versus the constant and asynchronous ventilation with continuous and uninterrupted chest compressions. To our knowledge, this is the first time, describing a cardiopulmonary resuscitation (CPR) of a preterm infant using HFOV.

## Case report

At the Medical University of Vienna, a tertiary perinatal center, a neonatal resuscitation team, consisting of at least four healthcare providers, attends deliveries of high-risk infants. During neonatal resuscitation of preterm infants < 32 gestational weeks, heart rate (HR), oxygen saturation on the right wrist, and blood pressure are measured.

### Maternal history and delivery

A 22-year-old primigravida delivered dichorionic diamniotic twins in 30^0/7^ weeks postmenstrual age. Pregnancy was complicated by a COVID-19 infection diagnosed in 28^0/7^ weeks’ and no gynecological control appointment had been scheduled during the 14-day quarantine period. Indication for C-section was given following a pathological Doppler ultrasound and intrauterine growth restriction of this twin without a premature rupture of membrane. Prior to delivery, the mother received one dose of antenatal steroids and magnesium sulphate. Our female patient, the first twin, was born with a birth weight of 970 g (14th percentile). The Apgar scores were 1/1/1 at 1, 5, and 10 min and the umbilical cord pH was 7.10. The second twin was not compromised after birth and had a favorable transition, clinical course, and short-term outcome.

**Fig. 1:**
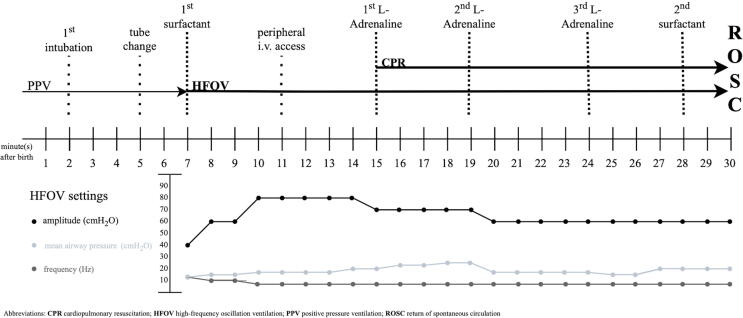
Resuscitation timeline with accompanied HFOV settings.

Resuscitation (displayed in Fig. 1).

The infant was born in her complete amniotic sac, which was opened at the resuscitation table. The newborn appeared floppy, cyanotic, and apneic with a HR slightly above 60/min. Positive pressure ventilation (PPV) was started approximately 30 s after birth using a facemask and T-piece device with the local default settings of 8 cmH_2_0 positive end-expiratory pressure and 25 cmH_2_O peak inflation pressure. Oxygen supply was initially set at 30% and due to the clinical situation quickly increased to 100%. Nasotracheal intubation was performed routinely due to failure of non-invasive ventilation in minute two with a 2.5 uncuffed endotracheal tube (ETT) to a depth of 8.0 cm. ETT placement was confirmed with laryngoscopy by two experienced neonatologists. Due to a loudly audible tube leak, it was changed to a 3.0 uncuffed ETT in minute five. No exhaled CO_2_ detection was used. Pulmonary auscultation indicated with hardly audible sounds poorly ventilated lungs and peak inflation pressure was increased to 40 cmH_2_O. Despite PPV and maximal oxygen supply, peripheral oxygen saturation remained unchanged at 50–60%. During resuscitation, the clinical team decided to use HFOV to try to open the lungs, because despite using high pressure levels with PPV, nearly no thorax excursions or breathing sounds were registered. HFOV was initiated at minute seven with the following settings: 12 cmH_2_O mean airway pressure (MAP), 40 cmH_2_O amplitude and 12 Hz frequency. Immediately after initiation of HFOV, surfactant (200 mg/kg, Curosurf®, Chiesi, USA) was administrated via the tube. It was decided, as a rescue approach, to continue HFOV, despite the dropping HR, and to open and recruit the lungs of this extremely low birth weight infant. To achieve lung recruitment, MAP was increased up to 24 cmH_2_O over seven minutes (Fig. 1). MAP was decreased afterwards to 16 cmH2O in order not to lower cardiopulmonary output and to avoid development of a pneumothorax, which was ruled out by diaphanoscopy.

**Fig. 2:**
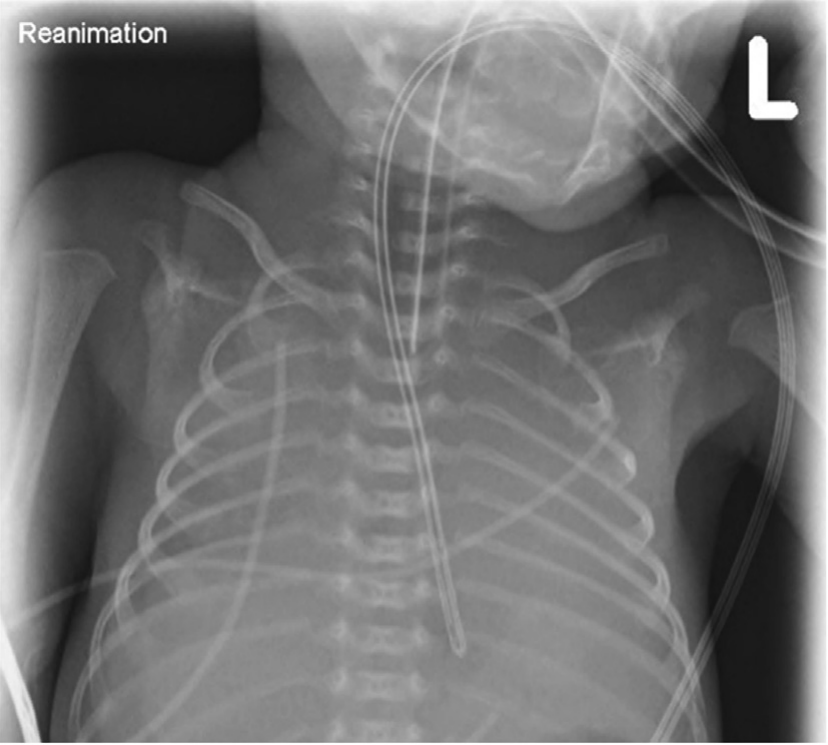
Chest x-ray during cardiopulmonary resuscitation, 18 min after birth.

At minute 11, a peripheral venous catheter was inserted, and the first blood pressure was measured with a mean of 27 mmHg. At minute 15, HR continuously dropped < 60/min and chest compressions were started using the two-thumb encircling technique at a rate of 120/min. Chest compressions were provided continuously, epinephrine (10 µg/kg) was administered at minute 15, 19, and 24, and a fluid bolus with 10 ml/kg isotonic solution was given immediately after the first dose of epinephrine. As shown in Fig. 2, the lungs of our patient had a bilateral white-out and were unaerated. Two minutes before the end of the 15-minutes-lasting CPR, a second dose of surfactant was administered followed by a second recruitment maneuver up to 20 cmH_2_O MAP, which obviously helped opening the lungs sufficiently to achieve ROSC. Afterwards, a rapid increase in HR was seen, which is considered the best indicator for adequate lung inflation.^1^

The blood pressures measured after CPR were in age-appropriate ranges.

The first venous blood gas taken from an umbilical vein catheter at minute 36 showed a pH 6.72, lactate 17 mmol/L, base excess −19.6 mmol/l, and pCO_2_ 115 mmHg.

### Clinical course

Lactate was below 2 mmol/L on the third day of life. Further, the patient was extubated after seven days and weaned of respiratory support on day 24 after birth.

On day two after birth, a cranial ultrasound revealed a bilaterally intraventricular hemorrhage grade 3 (as per Papile et al.,^2^ which did not result in posthemorrhagic hydrocephalus or EEG-abnormalities. A cranial MRI performed at term-equivalent age showed no evidence of asphyxia and was except for the intraventricular hemorrhage unremarkable.

The patient had an uneventful further clinical course and was discharged on day 70 of life (40^0/7^ weeks’).

## Discussion

The main cause of cardiovascular collapse in newborn infants is asphyxia due to failure of respiratory adaptation, which makes neonates different from the adult population.^3^ In preterm infants the adequate ventilation of their immature lungs, as well as securing the vulnerable airway, are some of the most important points during postnatal stabilization, whereas a failure in ventilation could eventually lead to CPR. It delineates the challenges a team deals within the resuscitation of preterm neonates.

The reason for the difficult resuscitation of this 30 weeks’ infant, with a birthweight on the 14th percentile without infection, was potentially lung hypoplasia following intrauterine growth restriction.^4^

The infant was intubated in minute two after birth following failure of non-invasive ventilation. A reason for that could have been laryngeal closure after birth.^5^ The optimal respiratory support during CPR with chest compressions is a major topic of many current research projects in neonatal resuscitation. To our knowledge, this is the first case report describing a successful neonatal resuscitation of a very preterm newborn using HFOV. Current resuscitation guidelines recommend to coordinate compression with ventilation (C:V) using a 3:1C:V ratio to avoid interference of chest compressions with tidal volume delivery.^1^ Several animal studies compared different C:V ratios: Pasquin et al^6^ reported that different C:V ratios resulted in similar ROSC and mortality rates in asphyxiated piglets. In resuscitation of asphyxiated piglets, using repeated 30 seconds sustained inflations superimposed by chest compression, significantly improved hemodynamics, minute ventilation, and time to ROSC compared to 3:1C:V ratio.7., 8., 9. Currently, the “SURV1VE” trial examines, if chest compressions superimposed with sustained inflation, compared to 3:1C:V, reduce time to ROSC and mortality in newborn infants.^10^

Another continuous form of ventilation, comparable to the principle of sustained inflations, is HFOV, which uses oscillation rates of 8–15 Hz with a more uniform lung inflation and thereby, it might reduce the severity of lung injury caused by intermittent PPV.^11^

Until now, no previous research has focused on HFOV during neonatal CPR, following the concept of sustained inflations with asynchronous chest compressions. HFOV adds a lung protective component, explained through low tidal volumes and a constant MAP in conjunction with high respiratory rates, and might help, combined with recruitment maneuvers, to improve gas exchange during CPR.12., 13. Altogether, our patient was without respiratory support at 34^0/7^ weeks gestational age, but had a severe intraventricular hemorrhage, which did not result in any neurological impairments in the first year of life.

## Conclusion

The presented case describes a rescue approach of ventilation during neonatal resuscitation using HFOV as the secondary ventilation mode resulting in ROSC. It highlights the importance of adequate lung inflation during CPR and might offer an alternative option to achieve this.

## Statement of Ethics, Written Informed Consent

Written informed consent was obtained from the parents for publication of the details of their medical case and any accompanying images.

## Funding Sources

The authors received no funding for the case report.

## Data Availability Statement

Additional anonymized medical record data are available from the Corresponding Authors upon reasonable request

## CRediT authorship contribution statement

**Julia Buchmayer:** Conceptualization, Methodology, Validation, Formal analysis, Resources, Data curation, Writing – original draft, Visualization, Project administration. **Lukas Wisgrill:** Data curation, Writing – review & editing. **Michael Schneider:** Data curation, Writing – review & editing. **Tobias Werther:** Methodology, Validation, Writing – review & editing, Supervision. **Katharina Goeral:** Writing – review & editing, Supervision. **Angelika Berger:** Writing – review & editing, Supervision. **Georg M. Schmölzer:** Conceptualization, Writing – review & editing. **Michael Wagner:** Conceptualization, Methodology, Validation, Formal analysis, Resources, Writing – review & editing, Project administration, Conceptualization, Methodology, Validation, Formal analysis, Resources, Writing – review & editing, Project administration.

## Declaration of Competing Interest

The authors declare that they have no known competing financial interests or personal relationships that could have appeared to influence the work reported in this paper.
